# Meta-Analysis of Milk Consumption and the Risk of Cognitive Disorders

**DOI:** 10.3390/nu8120824

**Published:** 2016-12-20

**Authors:** Lei Wu, Dali Sun

**Affiliations:** 1 Department of Epidemiology, Institute of Geriatrics, Chinese People’s Liberation Army General Hospital, Beijing 100853, China; 2 Department of Nanomedicine, Houston Methodist Research Institute, Houston, TX 77096, USA; DALI.SUN@asu.edu

**Keywords:** milk intake, Alzheimer’s disease, dementia, cognitive disorders, cognitive decline, meta-analysis

## Abstract

The association between milk intake and cognitive disorders has been investigated in several epidemiological studies, but the findings are still conflicting. No quantitative assessment has been performed to evaluate the potential relationship of milk intake and cognitive disorders. From the inception to October 2016, the PubMed and the Embase databases were searched for observational studies reporting the association of milk consumption and cognitive disorders (Alzheimer’s disease, dementia, and cognitive decline/impairment). A generic inverse-variance random-effects method was used to pool the Odds Ratios (ORs) and corresponding 95% confidence intervals (CIs) for the highest compared with the lowest level of milk intake. Subgroup and meta-regression analyses were used to assess the heterogeneity between subgroups. We identified seven articles involving a total of 10,941 participants. The highest level of milk consumption was significantly associated with a decreased risk of cognitive disorders, and the pooled OR (95% CI) was 0.72 (0.56, 0.93), with evidence of significant heterogeneity (*I*^2^ = 64%, *p* = 0.001). Subgroup analysis indicated that the association was more pronounced in ischemic stroke patients based on a single study. Furthermore, the inverse association between milk intake and cognitive disorders was limited to Asian subjects, and the African populations showed an intermediate non-significant trend. Although we have obtained a significant association, an established relationship cannot be drawn due to the study limitation. Large prospective studies are needed to quantify the potential dose-response patterns of milk intake and to explore the association in populations with different characteristics.

## 1. Introduction

Cognitive decline has become a major health problem with the aging of the population worldwide [1]. By 2050, the total estimated prevalence of Alzheimer’s disease (AD) is expected to be 13.8 million, whereas the proportion of death resulting from AD is still increasing [2]. Considering the undiscovered precise etiology of and the limited therapy of cognitive disorders, interventions on modifiable factors that may slow or prevent the development of cognitive decline have become an important public health issue.

A substantial amount of evidence has indicated that nutrition is associated with longevity and age-related diseases. Several healthy eating patterns and individual nutrients have shown preventive effects on cognitive decline [3,4,5,6]. In recent years, accumulating epidemiological studies have investigated the potential role of milk, which is one of the most popular nourishments around the world. The associations of milk or dairy intake and chronic diseases have been under investigation, but the results are not conclusive. Previous studies reported that milk intake was inconsistently associated high blood pressure, type II diabetes, cardiovascular disease (CVD), and mortality [7,8,9,10,11,12].

Milk and dairy foods have been inversely linked with the risk of stroke [13,14]. Hemorrhagic stroke is a potential risk factor of dementia; as a result, milk is also likely to be associated with dementia. In fact, previous epidemiological studies have explored the association of milk intake and cognitive disorders [15,16,17,18,19,20,21], but the conclusions remain contradictory. Some researchers have reported that the increased risk of cognitive disorders were significantly associated with lower intake of milk [15,16,17,18]; however, other studies did not corroborate the inverse association [19,20,21]. To the best of our knowledge, no quantitative assessment has been performed to evaluate the potential association between milk intake and cognitive disorders. Therefore, we conducted a systematic review and meta-analysis to summarize the evidence from observational studies on the relationship of milk consumption with the risk of age-related or vascular cognitive disorders (AD, dementia, cognitive decline, and cognitive impairment). Additionally, we performed subgroup and meta-aggression analyses to clarify whether the associations differed according to study characteristics.

## 2. Materials and Methods

### 2.1. Literature Search

We conducted the present systematic review and meta-analysis following the standard guidelines [22,23]. From the inception to October 2016, the PubMed and the Embase databases were searched for observational studies reporting the association of milk consumption and cognitive disorders. Our search was limited to studies written in English. Search terms included “dairy”, “milk”, “yogurt”, “AD”, “dementia”, “Alzheimer*”, “aphronesia”, “cognitive*” and “cognition” (Table A1 in Appendix A). We manually searched the reference lists of the relevant articles to identify more potential articles. When multiple publications from the same study were identified, the article with the longest follow-up duration was included.

### 2.2. Selection Criteria and Data Extraction

Initial screening was independently administered by two authors. After removing the duplicate articles, the title and abstract of each article was searched. Each article was independently assessed as exclusion or requiring further assessment. Disagreements were resolved by discussion within the two authors.

Inclusive criteria: (1) studies reporting relative risks (RRs) or hazard ratios (HRs) or odds ratios (ORs) and their corresponding 95% confidence intervals (CIs) of the cases of cognitive disorders involving intake of milk; (2) studies including a quantitative assessment of milk intake or milk combined other dairy products; (3) studies in which participants are aged ≥18 years old. Articles were excluded if (1) the data described a dietary pattern indicating a higher intake of milk, but did not describe the specific levels of milk intake; (2) the data described surrogate nutrients of milk, such as protein or fat; (3) the data described non-animal milk, such as breast milk or soy milk.

Data extraction was independently performed by two authors. The following data were extracted from each article: the first author and published year, study design, study location, number of participants and outcome cases, race, sex, the baseline age of participants, the duration of follow-up, the measurement method, the type of exposure and outcome, the category of exposure, adjustment variables, and the largest adjusted RRs, HRs, or ORs with corresponding 95% CIs of the risk of cognitive disorders. 

### 2.3. Quality Assessment

Quality assessment was independently performed by two authors. We used a guideline of Methodological Evaluation of Observational Research [24] and a quality assessment tool of observational cohort and cross-sectional studies [25] to evaluate the study quality of the eligible articles. Five domains were assessed for each article: design bias, selection bias, information bias, confounding, and analysis bias. The highest score of the five domains were 3, 4, 5, 3, and 2 points, respectively. The total scale scored from 0 to a possible maximum of 17 points, and a higher point indicated a higher study quality. Disagreements were resolved through consensus.

### 2.4. Statistical Analysis

Stata, version 12.0 (StataCorp LP, College Station, TX, USA), and the Review Manager, version 5.2 (The Nordic Cochrane Centre, Copenhagen, Denmark), were used to perform the statistical analyses. ORs (95% CIs) for the highest level of milk intake compared with the lowest were used to measure the effect sizes for articles that reported the outcome of cognitive disorders. A generic inverse-variance random-effects method was used to pool the outcome data. A two-sided *p*-value of less than 0.05 was considered significant. 

Q test and I^2^ statistic were used to evaluate the between-study heterogeneity, an I^2^ statistics of 50% or above was judged as statistically significant. Subgroup analysis was performed based on pre-specified characteristics: race (African, Asian, or Caucasian), gender (male, female, or both sexes), study design (cross-sectional or cohort), type of intake (milk or milk combined with other dairy products), the exposure assessment method (food-frequency questionnaire or others), and the type of participants (general population or patients). Meta-regression analysis was used to assess the heterogeneity between subgroups, and *p-*values of less than 0.1 were considered significant results. By omitting one article at every turn, sensitivity analysis was used to evaluate the influence of a single article on the overall pooled results. Publication bias was estimated through Begg and Egger’s tests [26,27].

## 3. Results

### 3.1. Study Identification and Selection

**Figure 1:**
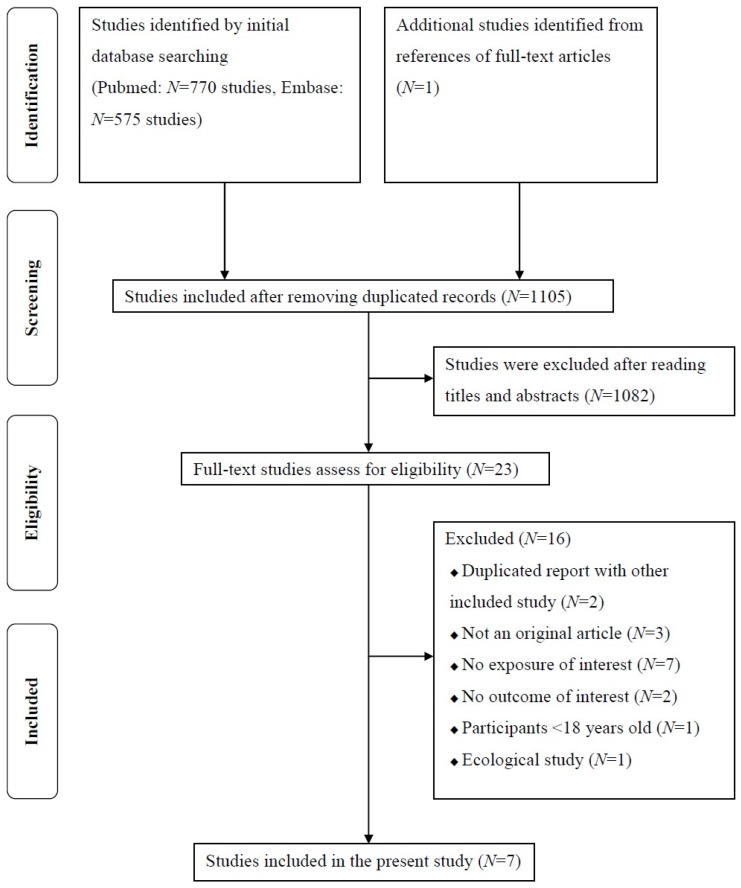
Flow diagram of articles included in the present study.

Detailed flow diagram of articles included in the present study is shown in Figure 1. A total of 1345 articles were identified from the Pubmed (770 articles) and the Embase (575 articles) databases. After removing duplicated articles, 1105 articles were included for further assessment. By reading the titles and the abstracts, 1082 articles were excluded. The remaining 23 full-text articles were further evaluated for eligibility. One additional record was identified from the reference of an article. Finally, a total of seven studies involving 12 comparatives were selected for the present systematic review and meta-analysis [15,16,17,18,19,20,21].

### 3.2. Study Characteristics

Table 1 presents the characteristics of each included study. These studies were published between 2006 and 2015. Three studies were performed in Caucasian [15,20,21], three in Asian [16,17,18], and one in African participants [19]. Among the cohort studies [15,16,18,21], the follow-up duration ranged between 4.8 and 25 years. Five articles included participants with both genders [16,17,18,19,20], one article included only females [15], and one included only males [21]. The sample size ranged from 601 to 4809 for a total of 10,941. The milk intake was assessed with a food-frequency questionnaire (FFQ) in two articles [16,19], and other studies used self-reported [15,17,20] or self-administrated questionnaire [18,21]. The participants of all included studies were of a healthy population, and the outcomes of all included studies were age-related cognitive disorders, except for one study [17]. Tu et al. explored the prevalence and effects of vascular cognitive impairment among ischemic stroke patients.

Cognitive disorders were diagnosed from DSM (Diagnostic and Statistical Manual of Mental Disorders) [16,18,19] for dementia; from NINCDS-ADRDA (National Institute of Neurological and Communicative Disorders and Stroke-Alzheimer’s Disease and Related Disorders Association) for Alzheimer’s disease [16,18]; and from MMSE (Mini-Mental State Examination) [15,17], Petersen criteria [19], a mental status questionnaire [20], or DECO (observed cognitive deterioration) [21] for cognitive impairment/decline.

### 3.3. Quality Assessment

The quality score of the seven included articles ranged from 12 [20] to 17 [16] points (Table A2 in Appendix A). The main quality issues were design bias, selection bias, and information bias. Three studies were based on cross-sectional design [17,19,20]. The withdraw rate of the four cohort studies were less than 20%. One study did not provide the information of eligibility criteria [20]. Two studies used the self-administrated questionnaire to measure the intake of milk [18,21].

### 3.4. Association between Milk Intake and Cognitive Disorders

**Figure 2:**
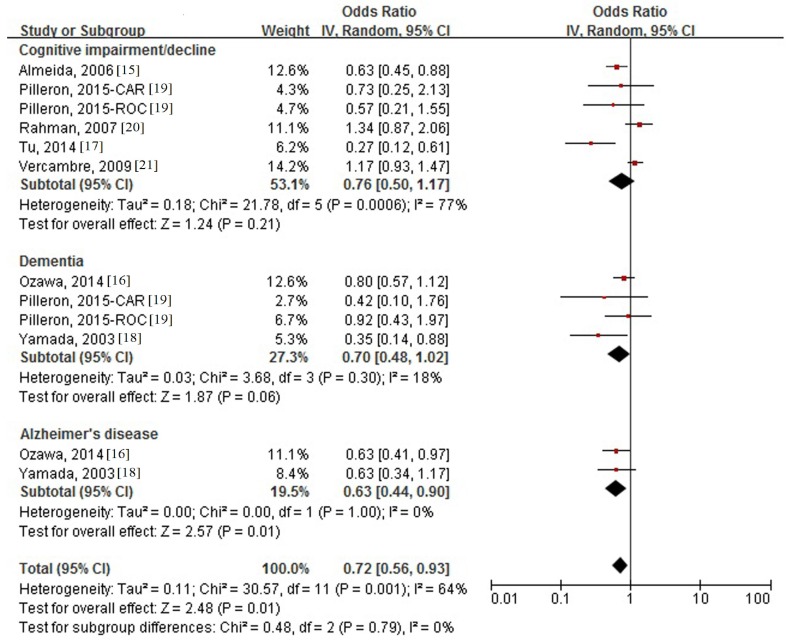
Forest plot of odds ratios (ORs) and 95% confidence intervals (CIs) for the association between milk intake and risk of cognitive disorders by type of outcome.

Seven studies comprising 12 comparatives reported the association between milk intake and the risk of cognitive disorders (Figure 2). Higher consumption of milk was significantly associated with the decreased risk of AD, and the pooled OR (95% CI) was 0.63 (0.44, 0.90), with no evidence of heterogeneity (*I*^2^ = 0%, *p* = 0.79). Non-significant and borderline significant results were shown relevant to the risk of cognitive impairment/decline (0.76 (0.50, 1.17)) and dementia (0.70 (0.48, 1.02)). The overall pooled OR (95% CI) of cognitive disorders was 0.72 (0.56, 0.93), with evidence of significant heterogeneity (*I*^2^ = 64%, *p* = 0.001). 

### 3.5. Subgroup Analysis and Meta-Regression

**Figure 3:**
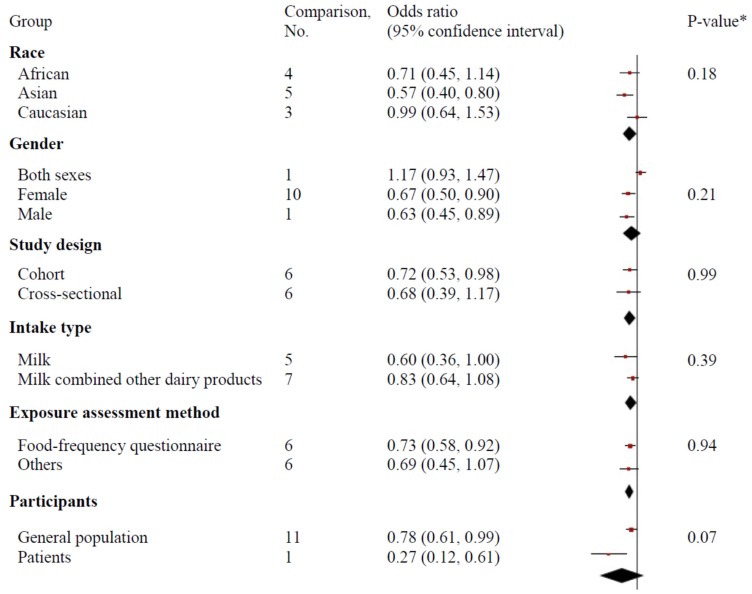
Subgroup analysis of the association between milk consumption and risk of cognitive disorders. * *p*-value for meta-regression.

As presented in Figure 3, analysis stratified by race, gender, study design, type of exposure, and exposure assessment method did not explain the heterogeneity between milk intake and cognitive disorders (*p*-value >0.1 for each subgroup). Subgroup analysis by the type of participants significantly affected the association, and the pooled ORs (95% CIs) were 0.78 (0.61, 0.99) for the general population and 0.27 (0.12, 0.61) for patients (*p*-value for difference = 0.07). 

### 3.6. Publication Bias and Sensitivity Analysis

**Figure A1:**
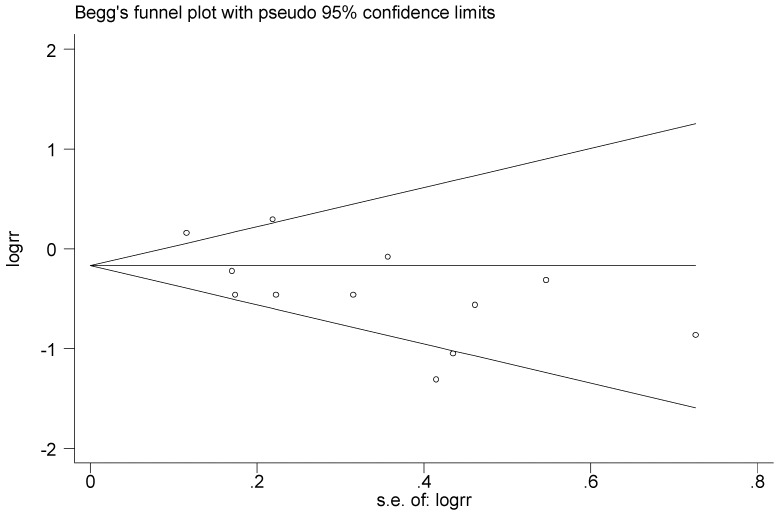
Funnel plot to explore the presence of publication bias.

As shown in Figure A1, visual inspection of the funnel plot did not suggest an evidence of publication bias among the articles (Egger’s test, *p* = 0.13; Begg’s test, *p* = 0.37). In the sensitivity analysis, exclusion of each article in every turn did not change the combined results, and the pooled ORs (95% CIs) ranged between 0.67 (0.51, 0.88) and 0.78 (0.62, 0.99).

## 4. Discussion

This systematic review and meta-analysis identified seven studies involving a total of 10,941 subjects. The combined analysis showed that milk intake was inversely associated with the risk of cognitive disorders. Compared with the lowest level of milk consumption, the risk of cognitive disorders was decreased by 28% with the highest level of milk consumption, but the result was based on limited number of studies with considerable heterogeneity.

Although the stratified analysis by race did not show significant group-difference in the three subgroups, there was some evidence that the inverse association between milk intake and cognitive disorders was limited to Asian subjects, and the African populations showed an intermediate non-significant trend. A clear difference in the amount of milk and dairy consumption among Asian and Western countries was reported [28]. Higher level of milk intake may significantly decrease the risk of cognitive disorders in populations (such as Asian) with relatively lower intake of overall milk and dairy products. In fact, excessive consumption of saturated fat in dairy products may adversely link with many chronic diseases [29], but low-fat dairy foods were reported to be associated with beneficial health outcomes [30,31]. Fat content was not described in all seven included studies, and only two studies reported more than three categories of milk intake; as a result, we could not separately evaluate the effect of whole-fat or low-fat milk. It is worth noting that the association was more pronounced in ischemic stroke patients based on a single study [17]. Tu et al. indicated that a higher level of milk consumption might be a potential preventive measure to reduce the prevalence of vascular cognitive disorders among ischemic stroke patients. However, limited to the cross-sectional nature and small sample size [17], false association should be considered. Further studies are still warranted to identify whether milk intake is more beneficial in some specific populations and diseases.

The preventive role of a diet rich in milk may be attributed to its protein, minerals, vitamins, and essential amino acids. Berg et al. reported that type 2 diabetes, hypertension, dyslipidemia, and obesity were associated with an increased risk of age-related cognitive dysfunction. Milk intake may reduce the risk of cognitive impairment via modifying neurovascular dysfunction, reducing weight and metabolic risks [32,33]. Animal studies also reported the effect of dairy products in anti-obesity [34,35]. Although no evidence from randomized controlled trials has investigated the preventive effect of milk consumption on cognitive disorders, several trials which showed the protective effect of milk intake against hypertension and obesity might partially support our finding [36,37,38].

This is the first systematic review and meta-analysis specifically evaluated the association between milk intake and cognitive disorders. We extended the work by Crichton et al., who quantitatively showed an inverse association between milk intake and cognitive disorders [39]. However, this study has several limitations. First, considerable heterogenicity was presented in our pooled analysis. We performed meta-regression analyses to investigate the possible explanation for the significant heterogeneity, and the results revealed that the heterogeneity might be associated with the characteristics of the subjects. Moreover, the diverse categories of milk intake (“times per week”, “gram per day”, “serving per day”, “high/low intake”, “tertiles”, etc.), the application of different dietary questionnaires, and different adjusted confounders may also lead to heterogeneous results. Second, we were unable to quantify the possible dose-response patterns of the association because of the different measurement units of milk consumption across studies. Furthermore, more than three categories of milk intake were not available in most of the included studies. High-quality prospective studies with standardized measurement unit should be administrated to further evaluate the potential dose-response effect of milk intake on the development of cognitive disorders. Third, because this is a meta-analysis based on observational studies, it is possible that the observed association is affected by unmeasured or residual confounding. For instance, two included studies only adjusted for a socio-demographic variable. A higher intake of milk may be linked with other healthy behaviors, such as regular physical exercise, a lower consumption of cigarettes and alcohol, and a lower intake of sugar and processed meat. All of the above behaviors were protective factors of cognitive disorders, but several included studies did not adjust for these possible confounding factors. Moreover, because of the nature of the cross-sectional design in most of the included studies, recall bias and selection bias cannot be avoided. Fourth, only two included studies used FFQ to measure the milk intake, and none of the studies corrected for measurement error. Fifth, whole-fat, low-fat, non-fermented, and fermented milk may have different associations with cognitive disorders; however, these details were poorly reported in the included studies. Finally, although we did not find publication bias in the statistical tests, having only seven included studies limits the interpretability of our results. Additional studies should assess the association between broader dietary patterns rather than sole ingredients (such as milk) and the risk of cognitive disorders with more confounding adjustments.

## 5. Conclusions

In summary, the present systematic review and meta-analysis of seven observational studies showed an inverse association between milk consumption and cognitive disorders. Although we obtained a significant association, an established relationship cannot be drawn due to the study limitations. Large prospective studies are needed to quantify the potential dose-response patterns of milk intake and to explore the association in populations with different characteristics.

## Figures and Tables

**Table 1:** Characteristics of included studies.

First Author, Year	Study Design	Country	Follow-Up (Years)	Male (%)	Baseline Age (Years)	Participants, No.	Exposure		Outcome			Adjustment *
							Method of Assessment	Category	Type	Method of Assessment	Case, No.	
Almeida, 2006 [15]	Cohort	Australia	4.8	100.0	65−	601	Self-report questionnaire	Rare, regularly	Cognitive impairment	MMSE < 24	144	1–4
Ozawa, 2014 [16]	Cohort	Japan	17	42.2	60−	1081	70-item semi-quantitative FFQ	Women: <45, 45–96, 97–197, ≥198 g/day; Man: <20, 20–75, 76–173, ≥174 g/day	Dementia, AD	DSM-III-R, NINCDS-ADRDA	303, 166 ^1^	1, 2, 4–16
Pilleron, 2015 [19] CAR; ROC	Cross-sectional	Africa	-	40.3; 41.4	65−	841; 931	8-item FFQ	<1 serving/day, ≥1 serving/day	Dementia, Cognitive impairment	DSM-IV, Petersen criteria	72, 62; 63, 56 ^2^	1, 2, 4, 17, 18
Rahman, 2007 [20]	Cross-sectional	USA	-	32.7	65−	1056	Self-report questionnaire	<1 time/week, ≥1 time/week	Cognitive impairment	Mental status questionnaire <9	175	1, 2, 4, 12–15
Tu, 2014 [17]	Cross-sectional	China	-	58.6	40−	689	Self-report questionnaire	Low, high intake	Cognitive impairment	MMSE < 28 and MoCA-CS < 27	221	1, 13, 16, 19–28
Vercambre, 2009 [21]	Cohort	France	13	0	63−	4809	Self-administrated questionnaire	Tertiles	Cognitive decline	DECO < 33	598	1, 2, 4, 6, 7, 9–11, 29-35
Yamada, 2003 [18]	Cohort	Japan	25	26.8	30−	1774	Self-administrated questionnaire	<4 times/week, daily	Dementia, AD	DSM-IV, NINCDS-ADRDA	114, 51 ^3^	1, 2, 7, 9, 36, 37

CAR: Central African republic; ROC: Republic of Congo; FFQ: food frequency questionnaire; AD: Alzheimer’s disease; MMSE: Mini-Mental State Examination; MoCA-CS: Montreal Cognitive Assessment-Changsha version; DSM: Diagnostic and Statistical Manual of Mental Disorders; NINCDS-ADRDA: National Institute of Neurological and Communicative Disorders and Stroke-Alzheimer’s Disease and Related Disorders Association; DECO: observed cognitive deterioration.* 1 = age; 2 = education; 3 = English-speaking background; 4 = physical activity; 5 = stroke; 6 = hypertension; 7 = diabetes mellitus; 8 = total cholesterol; 9 = BMI; 10 = smoking habits; 11 = energy; 12 = vegetable; 13 = fruit; 14 = fish; 15 = meat intake; 16 = education; 17 = area; 18 = marital status; 19 = occupation; 20 = Aconuresis; 21 = Paraventricular WML; 22 = Macroangiopathy; 23 = alcohol intake; 24 = regular health checks; 25 = having a hobby; 26 = sleep time; 27 = nap habit; 28 = dietary structure; 29 = supplement consumption; 30 = use of postmenopausal hormones; 31 = hypercholesterolemia; 32 = CHD; 33 = stroke; 34 = cancer; 35 = depression; 36 = SBP; 37 = eating with salt or soy sauce. ^1^ Number of dementia cases and cognitive impairment cases, respectively; ^2^ Number of dementia cases and cognitive impairment cases of each cohort separated by semicolon; ^3^ Number of dementia cases and Alzheimer’s disease cases, respectively.

## Appendix A

**Table A1:** Search strategy.

**Source: PubMed (Searched on: 30 October 2016)**
#1 dairy [Title/Abstract]
#2 milk [Title/Abstract]
#3 yogurt [title/abstract]
#4 dementia [title/abstract]
#5 AD [title/abstract]
#6 Alzheimer* [title/abstract]
#7 aphronesia [title/abstract]
#8 cognitive* [title/abstract]
#9 “humans” [MeSH Terms]
#10 English [lang]
#11 #1 OR #2 OR #3
#12 #4 OR #5 OR #6 OR #7 OR #8
#13 #9 AND #10 AND #11 AND #12
**Source: Embase (Searched on: 30 October 2016)**
#1 dairy:ti,ab
#2 milk:ti,ab
#3 yogurt:ti,ab
#4 dementia:ti,ab
#5 AD:ti,ab
#6 Alzheimer*:ti,ab
#7 aphronesia:ti,ab
#8 cognitive*:ti,ab
#9 “human“/de
#10 #1 OR #2 OR #3
#11 #4 OR #5 OR #6 OR #7 OR #8
#12 #9 AND #10 AND #11

**Table A2:** Quality assessment of each eligible article (maximum score = 17).

First Author, Published Year	Design Bias	Selection Bias	Information Bias	Confounding	Analysis Bias	Total
Almeida, 2006 [15]	3	4	4	3	2	16
Ozawa, 2014 [16]	3	4	5	3	2	17
Pilleron, 2015 [19]	1	3	5	3	2	14
Rahman, 2007 [20]	1	2	4	3	2	12
Tu, 2014 [17]	1	3	4	3	2	13
Vercambre, 2009 [21]	3	4	4	3	2	16
Yamada, 2003 [18]	3	4	4	3	2	16

Selection column includes five items: (1) Design bias: study design and follow up; (2) Selection bias: inclusion/exclusion criteria, recruitment strategy, interval between exposure & outcome assessment, and attrition; (3) Information bias: pre-specified outcome, outcome assessment, reliability of the outcome, data collection & assessment, and exposure assessment; (4) Confounding: confounding adjustment; and (5) Analysis bias: effect size, and data availability.
